# A Clinical Nomogram for Predicting Lymph Node Metastasis in Penile Cancer: A SEER-Based Study

**DOI:** 10.3389/fonc.2021.640036

**Published:** 2021-03-09

**Authors:** Wei Zhang, Pan Gao, Jingjing Gao, Xu Wu, Guodong Liu, Xiansheng Zhang

**Affiliations:** Department of Urology, The First Affiliated Hospital of Anhui Medical University, Hefei, China

**Keywords:** penis (MeSH), carcinoma, squamous cell, lymph nodes, nomograms

## Abstract

**Purpose:** We developed a nomogram to predict the possibility of lymph node metastasis in patients with squamous cell carcinoma of the penis.

**Methods:** Identifying patients with squamous cell carcinoma of the penis diagnosed between 2004 and 2015 in the Surveillance, Epidemiology, and End Results (SEER) database. Univariate and multivariate analyses were carried out by logistic regression to assess significant predictors associated with lymph node metastasis. A nomogram was established and validated by a calibration plot and receptor operating characteristic curve (ROC) analysis.

**Results:** A total of 1,016 patients with penile squamous cell carcinoma (SCCP) were enrolled in this study. One hundred and ninety-five patients (19%) had lymph node involvement (N1-3). Multivariate analysis showed that age, primary tumor site, grade, tumor size, and T stage were identified as being significantly (*p* < 0.05) associated with lymph node involvement. All the above factors that showed a statistically significant predictive capability were selected for building the nomogram. This model had a calibration slope of 0.9 and a c-index of 0.776, indicating the good discrimination and effectiveness of the nomogram in predicting lymph node status.

**Conclusion:** Although the prediction model has some limitations, the nomogram revealed the relationship between the clinicopathological characteristics of SCCP patients and the risk of lymph node metastasis. This tool will assist patients in counseling and guide treatment decisions for SCCP patients.

## Introduction

Penile cancer is a rare malignant tumor of the genitourinary system, accounting for <0.1% of all malignancies in men living in the developed world, while its incidence rates are higher in parts of South America and Africa (1). Squamous cell carcinoma is the most common histology of penile cancer, accounting for more than 95% (2), and commonly occurs in men between 50–70 years old (3). Besides, 80% of the primary tumors are localized at the glans and prepuce (4).

Lymph node metastases of squamous cell carcinoma of the penis (SCCP) affects the selection of surgical therapy and is also a strong predictor of prognosis, patients with lymph node metastases were proven to have a worse prognosis (5). About 80% of men with low-grade penile cancer can achieve prolonged survival, but as the degree of lymph node metastasis increases, the survival rate decreases precipitously (6, 7). The 5-year survival of patients with inguinal lymph node (ILN) metastasis can be as high as 80%, while patients with pelvic lymph node (PLN) metastasis and distant metastases have a survival rate of 0–33% (8, 9). Early metastatic spread to regional lymph nodes can be life-threatening (10).

Because of the high possibility of lymph node dissection, it is very important to determine the appropriate surgical candidate. However, few studies to date have evaluated the risk factors or predictive models of lymph node metastases. Ficarra et al. (11) formed the first nomogram to predict lymph node involvement based on a cohort of 265 patients. The clinical stage of the inguinal lymph node, histological grade, and other tumors pathological features w included in the model, and multivariate analysis showed that only lymphovascular invasion and clinically palpable lymph nodes were significant predictors of lymph node status. Velazquez et al. (12) later developed a more specific nomogram to predict lymph node metastasis, found that perineural infiltration and grade were significant predictors. Also, Bhagat et al. (13) demonstrated that age, tumor grade, lymphatic vascular infiltration, and clinically palpable lymph nodes were predictors of lymph node involvement. However, the tumor stage had not proven to be significant which is analogous to some other research (12, 14). Recently, a cohort study including 380 penile cancer patients between 2000 and 2010 was implemented to identify predictors of lymph node involvement, multivariable analysis demonstrated that age, pathological stage, tumor grade were independently associated with lymph node involvement. Moreover, the accuracy tests of the risk stratification scheme suggested that there were no significant differences between different risk group systems (15). The result is still controversial. It is worth noting that in terms of demographics and clinicopathological information, there is great heterogeneity among SCCP patients, such as age, race, marital status pathological type, tumor size, and primary tumor site (16). Therefore, a well-designed predictive model for lymph node metastases in SCCP patients covering more factors is needed. This study aimed to identify clinical and pathology characters of SCCP, to predict lymph node metastases of non-metastatic (M0) squamous cell carcinoma of the penis, then construct and validate a novel nomogram for predicting lymph node metastases in M0 SCCP using a cohort from the Surveillance, Epidemiology, and End Results (SEER) database.

## Methods

### Patients and Selection Criteria

This retrospective study analyzed the data of patients with squamous cell carcinoma of the penis diagnosed between 2004 and 2015, extracted from the Surveillance, Epidemiology, and End Results (SEER) database (accession number is 15779-Nov2019). Incomplete records on primary tumor site, grade, TNM stage, marital status, tumor size were excluded from the study. and non-squamous cell carcinoma [According to the “International Classification of Diseases-Oncology, 3rd edition” (ICD-O-3), the code of squamous cell carcinoma of the penis was 8051–8052 and 8070–8075 (17)] and patients with distant metastasis were also not included. Patients were excluded if they underwent any type of neoadjuvant therapy (including radiation, chemotherapy, hormone, therapy, or other systemic therapy). All the patients we included underwent surgical treatment, including partial penectomy, total penectomy, and organ sparing surgery. And lymph node staging was identified through surgery. The demographic variables of marital status at diagnosis, age at diagnosis, race and primary tumor site, tumor characteristics of differentiation grade, histological type, T stage, N stage, and tumor size were collected from the SEER database using SEER-stat software.

TNM stages of the penile tumor were determined according to the American Joint Committee on Cancer (AJCC) 6th edition staging system using available clinical and pathologic data on tumor invasion, lymph nodes status, and distant metastasis, respectively. The definitions are as follows: T1 is defined as a tumor invading subepithelial connective tissue; T2 is defined as a tumor invading corpus spongiosum with or without invasion of the urethra; T3 is defined as a tumor invading corpus cavernosum with or without invasion of the urethra; T4 is defined as a tumor invading other adjacent structures; N0 means no palpable or visibly enlarged inguinal lymph nodes; N1 means palpable mobile unilateral inguinal lymph node; N2 means palpable mobile multiple or bilateral inguinal lymph nodes; N3 means fixed inguinal nodal mass or pelvic lymphadenopathy, unilateral or bilateral; M0 means no distant metastasis; and M1 means distant metastasis. The histopathological grading of penile carcinoma was determined according to the SEER cancer grade system. Data of marital status at diagnosis, age at diagnosis, race, and tumor size were divided into different groups after being processed.

The SEER database is a public database and has patient anonymization, the use of a public database without patient identification information meets the requirements of the institutional review board and the ethics committee.

### Statistical Analysis

Statistical analyses to identify prediction factors were performed using SPSS 15.0 for Windows (SPSS, Chicago, IL). The Chi-square-test was used to determine the significance of differences between categorical variables. Some variables such as tumor size were grouped based on the median of the overall data. Univariate and multivariate analyses were carried out by logistic regression, and odds ratios (ORs) and 95% confidence intervals (CIs) were calculated. All reported *p*-values were two-sided, and a *p*-value of <0.05 was considered statistically significant.

A mosaic plot was constructed to show the distribution and relationship of clinicopathological characteristics of SCCP patients by using the package of vcd in R version 2.14.1 (http://www.r-project.org/). Nomograms from multivariable logistic models are a popular visual plot to display the predicted probabilities of an event for decision support (18). A nomogram was formulated based on the results of multivariate analysis and by using the package of rms in R version 2.14.1 (http://www.r-project.org/), to predict lymph node metastases in M0 squamous cell carcinoma of the penis. To test the performance of the nomogram, it was subjected to 1000 bootstrap resamples for internal validation to calculate the corrected c-index. A calibration curve was created using the observed lymph node status and the predicted lymph node status. Moreover, the ROC curve was used to evaluate the effectiveness of the nomogram.

## Results

### Patient Characteristics

**Figure 1:**
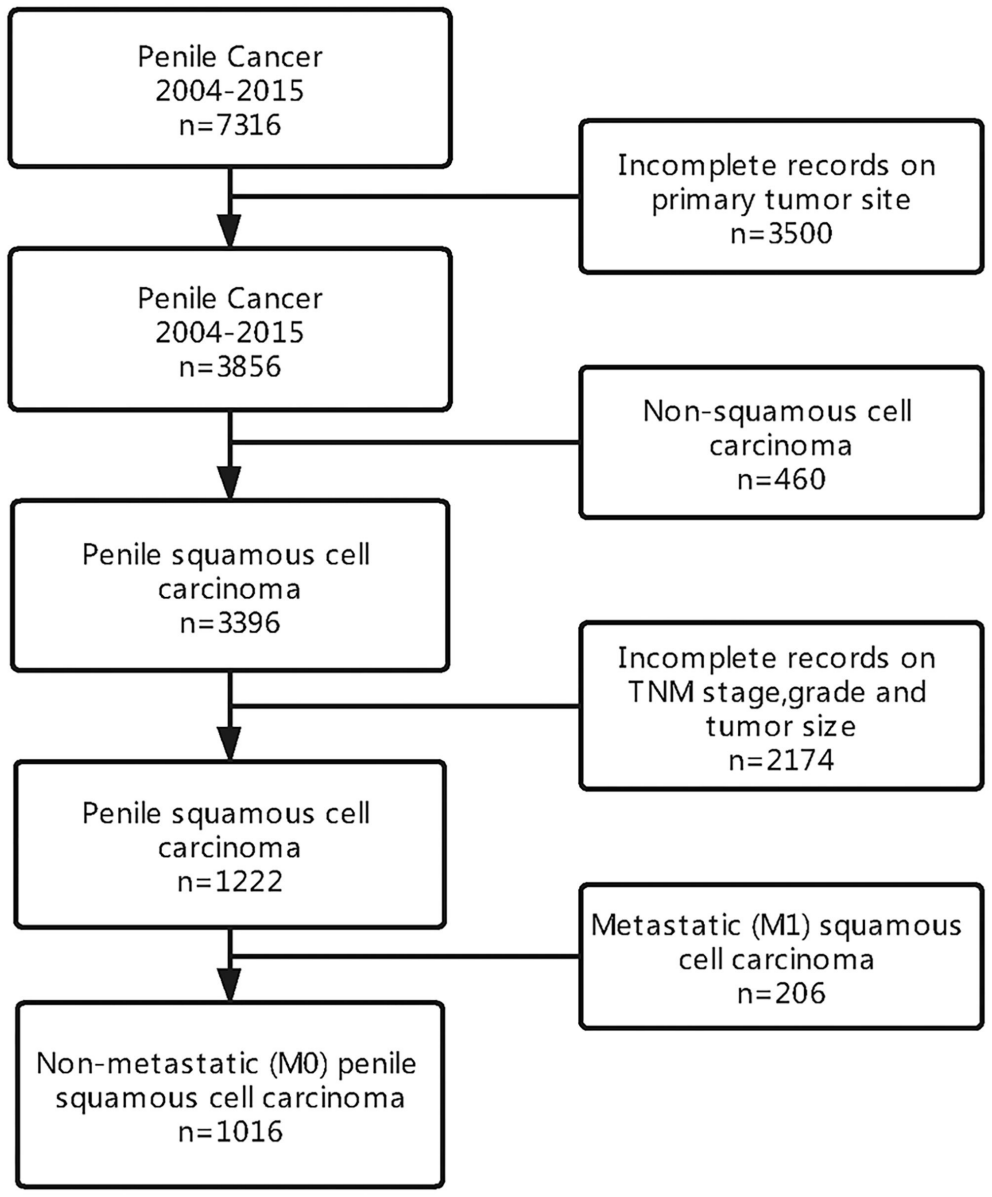
The flow diagram of patient selection.

We identified a cohort of men with penile squamous cell carcinoma from the SEER database. Of the 7,316 patients diagnosed with penile cancer, a total of 1,016 men were included in our analysis. A summary of study selection criteria can be seen in Figure 1.

A total of 1,016 patients with penile non-metastatic (M0) squamous cell carcinoma were enrolled in this study, and the specific tumor site was identified in all patients. Among the 1,016 included patients, 195 (19%) had lymph node involvement (N1-3) and 821 (81%) had N0 status. The median age of patients was 60 years, and the mean tumor size was 3 cm. The majority of patients where white (855, 84.2%), with a significantly smaller percentage of men being black (96, 9.4%) or other (65, 6.4%). Most of the cases were married (703, 69.2%). Patients characteristics and the association of lymph node status with demographic characteristics and clinicopathological characteristics were presented in Table 1.

**Table 1:** Comparison of demographics and clinicopathological characteristics of patients with different lymph nodes status.

**Demographics and clinicopathologic characteristics**	**Patients without lymph node metastases (N0 stage)**		**Patients with lymph node metastases (N1-3 stage)**		***P***
	**No. of patient (*n* = 821)**	**%**	**No. of patient (*n* = 195)**	**%**	
Age					0.004
<50 years	96	11.7%	41	21.0%	
50–69 years	358	43.6%	90	46.2%	
≥70 years	367	44.7%	64	32.8%	
Race					0.867
White	693	84.4%	162	83.1%	
Black	77	9.4%	19	9.7%	
Other^a^	51	6.2%	14	7.2%	
Marital status					0.002
Married	586	71.4%	117	60.0%	
Unmarried^b^	235	28.6%	78	40.0%	
Primary site					0.011
Prepuce	166	20.2%	23	11.8%	
Glans penis	525	63.9%	127	65.1%	
Body of penis	68	8.3%	25	12.8%	
Overlapping lesion of penis	62	7.6%	20	10.3%	
Tumor size					<0.001
<3 cm	434	52.9%	69	35.4%	
≥3 cm	387	47.1%	126	64.6%	
Grade					<0.001
I	260	31.6%	20	10.3%	
II	404	49.2%	97	49.7%	
III	154	18.8%	77	39.5%	
IV	3	0.4%	1	0.5%	
T-stage					<0.001
T1	438	53.3%	37	19.0%	
T2	255	31.1%	81	41.5%	
T3	127	15.5%	71	36.4%	
T4	1	0.1%	6	3.1%	

a *Includes: American Indian/native Alaskan and Asian/Pacific Islander*.

b *Includes: divorced, separated, single, domestic partner and widowed*.

There were no significant differences in the race category by lymph node status. And there was a statistically significant difference in age, marital status, the primary site of the tumor, tumor size, different grade of differentiation, and T stage between the patients with lymph node involvement and those without (*p* < 0.05 for all).

### Comparison of Oncology Features of Patients With Different Primary Tumor Site

**Figure 2:**
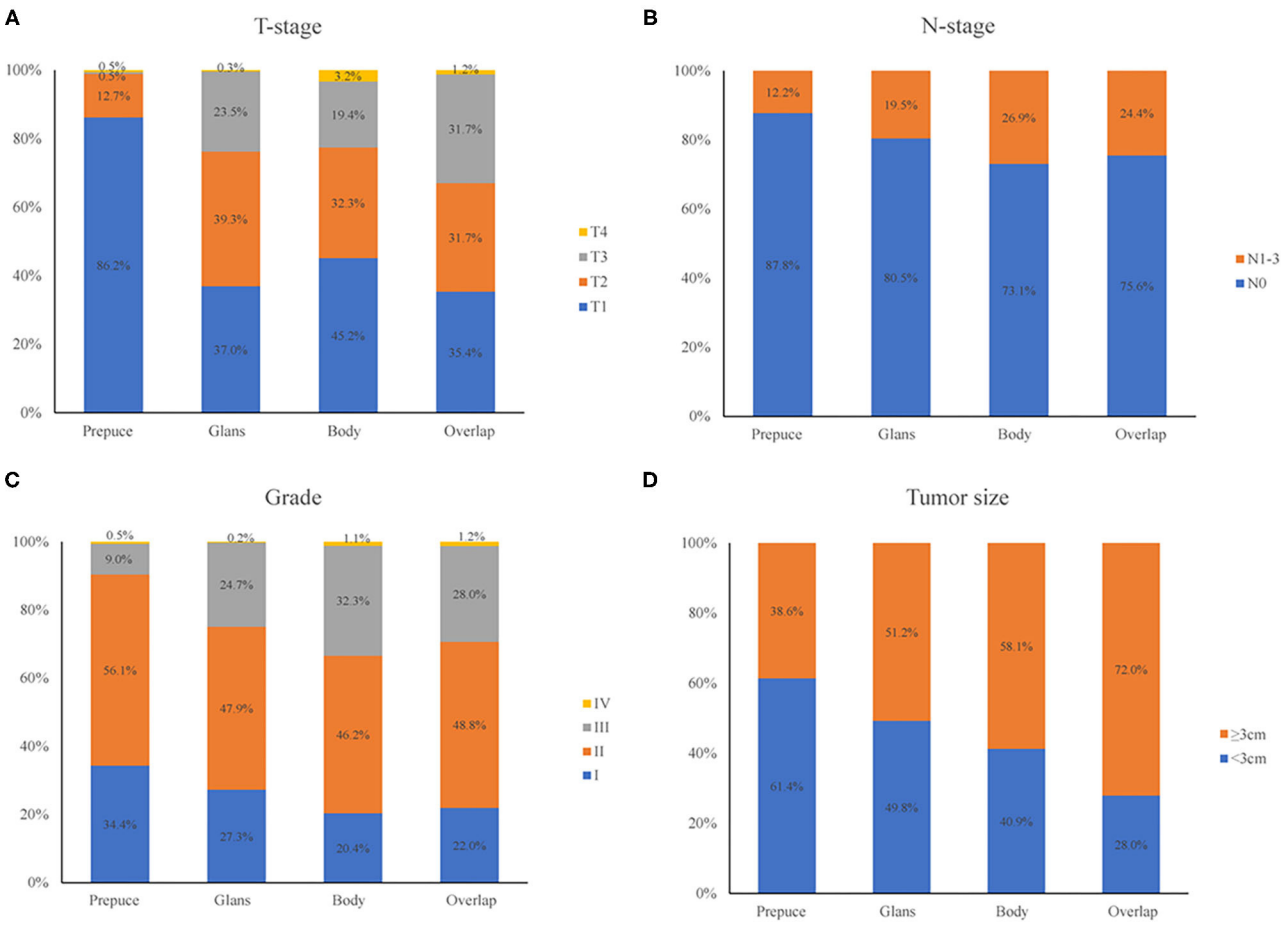
Comparison of oncology features of patients with different primary tumor site. T-stage **(A)**, N-stage **(B)**, tumor grade **(C)**, and tumor size **(D)** had different distribution in patients with different primary tumor site (all *p* < 0.001).

In this group of patients, tumors occurred in the prepuce, glans, the body of the penis, and some were overlapping lesions, the numbers were 189 (18.6%), 652 (64.2%), 93 (9.1%), and 82 (8.1%), respectively. Patients were grouped concerning their primary tumor site and compare the oncology features of each group, the result was shown in Figure 2. T stage, N stage, tumor grade, and tumor size had a different distribution in patients with different primary tumor site (all *p* < 0.001). Most patients with primary tumors that localized at the prepuce were in T1 and T2 stage, and a higher proportion of tumors with overlapping lesions were in stage T3 compared with those localized at other sites (Figure 2A); Compared with primary tumors that localized at the prepuce, tumors that localized at the body of the penis had a higher probability of lymph node involvement (Figure 2B); Compared with tumors located in other sites, tumors with overlapping lesions or localized at the body of the penis had a worse differentiation grade (Figure 2C); Compared with tumors located in other sites, tumors with overlapping lesions had a larger tumor size (Figure 2D); In general, the primary tumor site was closely related to the pathological characteristics of the tumor.

### Distribution and Relationship of Clinicopathological Characteristics in Patients With SCCP

**Figure 3:**
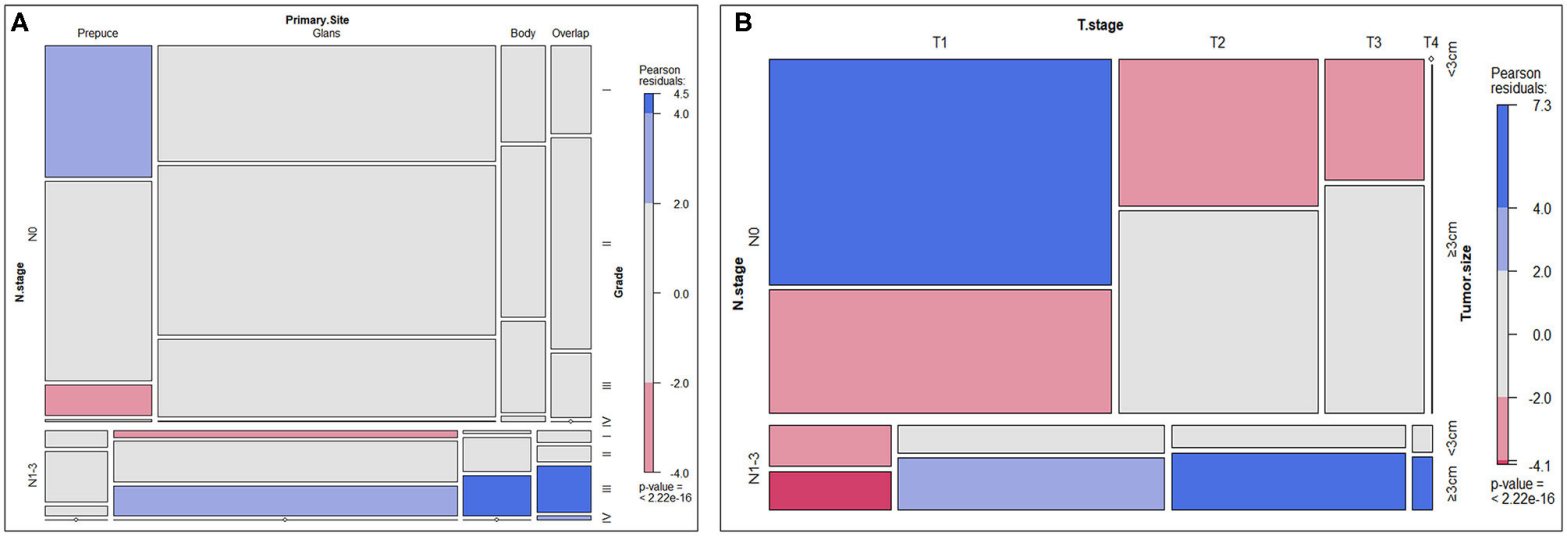
Mosaic plot. **(A)** Distribution and relationship of N-stage, primary site, and tumor grade. **(B)** Distribution and relationship of N-stage, T-stage, and tumor size.

A mosaic plot was applied to show the distribution and relationship of clinicopathological characteristics of SCCP patients. In the mosaic plot, the area of the nested matrix is proportional to the cell frequency, where the frequency is the frequency in the multi-dimensional contingency table. The color and shading can indicate the residual value of the fitted model. Patients with lymph node involvement (N1-3) had higher tumor grade, more advanced clinical tumor stage, larger tumor size, and its primary tumor site was also significantly different from patients without lymph node involvement (N0). The result was shown in Figure 3.

### Univariate and Multivariate Analyses and Identification of Predictive Factors

Binary classification logistic regression was applied to investigate the predictive factors associated with lymph node metastases in patients with M0 SCCP. The results of the univariate analysis showed that age, marital status, primary tumor site, grade, tumor size, and T stage were identified as being significantly (*p* < 0.05) associated with lymph node involvement (N1-3). Further multivariate analysis showed that younger age [(<50 years vs. 50–69 years, OR 0.534, 95% CI, 0.331–0.863), (<50 years vs. ≥70 years, *p* = 0.01 OR 0.357, 95% CI, 0.215–0.594), *p* < 0.0001], larger tumor (OR 1.461, 95% CI, 1.017–2.098, *p* < 0.0001), higher grade [(II vs. I, OR 2.84, 95% CI, 1.679–4.805), (III, IV vs. I, OR 5.629, 95% CI, 3.201–9.900), *p* < 0.0001] and T stage [(T2 vs. T1, OR 3.717, 95% CI, 2.306–5.991), (T3, T4 vs. T1, OR 6.173, 95% CI, 3.677–10.362), *p* < 0.0001] were high risk factors of lymph node involvement. Moreover, the primary tumor was localized in the body of the penis (OR 1.456, 95% CI, 1.013–1.565, *p* = 0.034), overlapping lesion of the penis (OR 1.613, 95% CI, 1.035–1.781, *p* = 0.023) were also predictors of lymph node metastasis in penile squamous cell carcinoma. A summary of the results was displayed in Table 2.

**Table 2:** Univariate and multivariate analysis of factors associated with lymph node metastases.

**Variables**	**Univariate analysis**			**Multivariate analysis**		
	**OR**	**95% CI**	**P**	**OR**	**95% CI**	***P***
Age						<0.001
<50 years	1	Reference	–	1	Reference	–
50–69 years	0.589	0.382–0.907	0.016	0.534	0.331–0.863	0.01
≥70 years	0.408	0.260–0.642	<0.001	0.357	0.215–0.594	<0.001
Race						
White	1	Reference	–			
Black	1.056	0.621–1.794	0.842			
Other^a^	1.174	0.634–2.173	0.609			
Marital status						
Married	1	Reference	–	1	Reference	–
Unmarried^b^	1.662	1.202–2.299	0.002	1.217	0.847–1.747	0.289
Primary Site						0.039
Prepuce	1	Reference	–	1	Reference	–
Glans penis	1.746	1.083–2.814	0.022	1.122	0.933–2.563	0.069
Body of penis	2.328	1.196–4.534	0.003	1.456	1.013–1.565	0.034
Overlapping lesion of penis	2.652	1.409–4.996	0.013	1.613	1.035–1.781	0.023
Tumor size						
<3 cm	1	Reference	–	1	Reference	–
≥3 cm	2.048	1.481–2.831	<0.001	1.461	1.017–2.098	<0.001
Grade						<0.001
I	1	Reference	–	1	Reference	–
II	3.121	1.882–5.177	<0.001	2.84	1.679–4.805	<0.001
III, IV	6.459	3.803–10.969	<0.001	5.629	3.201–9.900	<0.001
T-stage						<0.001
T1	1	Reference	–	1	Reference	–
T2	3.760	2.475–5.714	<0.001	3.717	2.306–5.991	<0.001
T3, T4	7.121	4.592–11.043	<0.001	6.173	3.677–10.362	<0.001

*OR, odds ratio; CI, confidence interval; “-” = no data*.

a *Includes: American Indian/native Alaskan and Asian/Pacific Islander*.

b *Includes: divorced, separated, single, domestic partner and widowed*.

### Construction and Validation of the Nomogram

**Figure 4:**
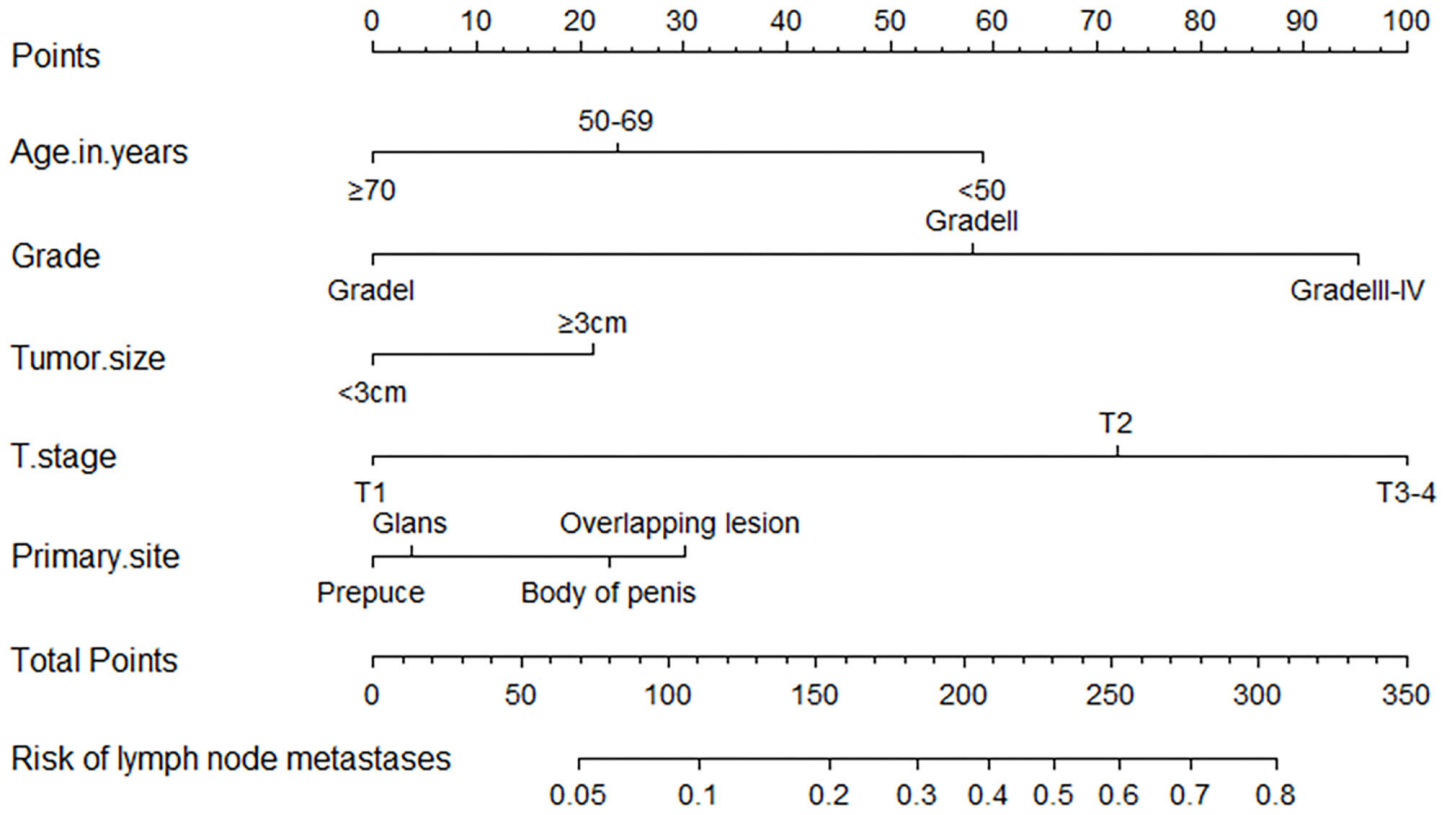
A nomogram for predicting the probability of lymph node metastases. To use the nomogram, the value for each predictor is determined by drawing a line upward to the point reference line, the points are summed, and a line is drawn downward from the total points line to find the predicted probability of lymph node metastases.

All the above factors that showed a statistically significant predictive capability for lymph node metastasis were selected for building the nomogram. The factors included in the final nomogram were age, primary tumor site, grade, tumor size, and T stage. In the nomogram, tumor grade and T stage have a more significant impact on the lymph node metastasis. Besides, age, primary tumor site, and tumor size also had varying degrees of influence on lymph node involvement, shown in Figure 4.

The process of using the nomogram model to individually predict the risk of lymph node involvement of a patient is as follows: (1) Determine the score of each predictor on the scale, (2) calculate the total score of 5 predictors, (3) draw a straight line from the total points line down to the bottom risk line to find the risk of lymph node involvement of a patient.

**Figure 5:**
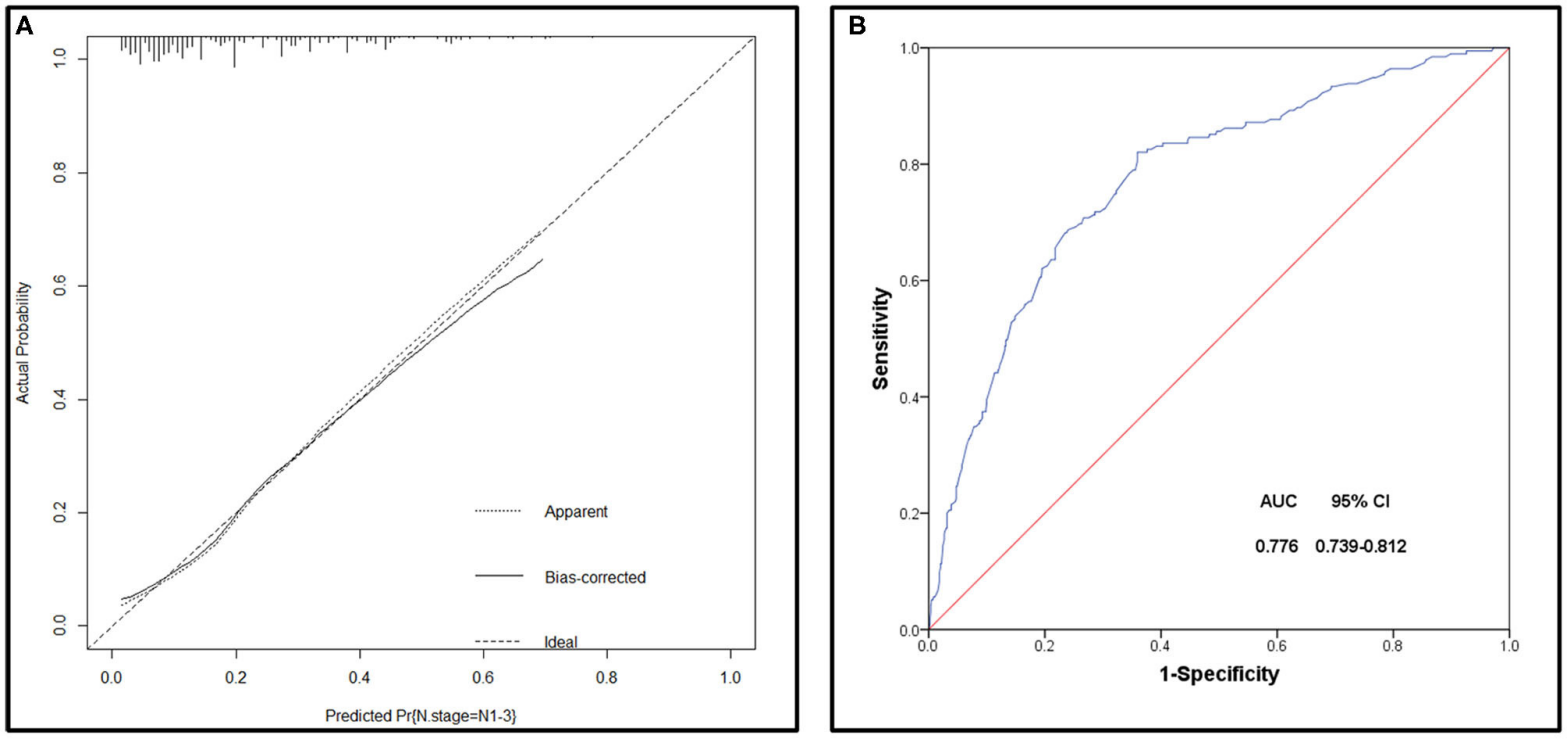
**(A)** Calibration plot of the nomogram for the probability of lymph node metastases (bootstrap 1,000 repetitions). **(B)** Receiving operating characteristic (ROC) curve of the prediction model.

The bootstrap method was applied to internally verify the prediction performance of the model, indicated that the model has better discrimination in predicting lymph node involvement in SCCP patients, and a calibration plot was forthput to assess the agreement between observed and predicted values, showed that this nomogram was well-calibrated (Figure 5A). Moreover, we evaluated the effectiveness of the nomogram in predicting lymph node metastasis by using the ROC curve (Figure 5B), according to Youden's method, optimal cutoff values of the nomogram were 0.189, and the sensitivity, specificity associated with the 0.189 cut-offs were 79.5 and 34.2%, respectively. According to the clinicopathological data of the studied cohort, we could assess the possible risk of lymph node involvement of patients with SCCP, and patients with the risk of lymph node invasion >0.189 were considered as a high-risk group, which it was recommended to perform a lymphadenectomy. In general, this model had a calibration slope of 0.9 and a c-index of 0.776, indicating the good discrimination and effectiveness of the nomogram in predicting lymph node status.

## Discussion

The lack of expertise of clinicians and the public disgrace among patients have created an environment where up to 25% of men have advanced disease at the time of diagnosis (19), and treatment is delayed for over 1 year in up to 50% of patients (20). Therefore, many patients with penile cancer have metastatic disease, the progression of metastasis follows a predictable gradual invasion pattern from the primary tumor to the inguinal lymphatic pool, then spread to the pelvic lymph nodes and systemic spread (21), which is a major prognostic factor for penile cancer survival and associated with poor prognosis (22). Approximately 70% of patients were metastatic lymph nodes among patients with at least one clinically palpable nodule (cN+) (22). In other cases, lymph node enlargement is caused by inflammation, usually secondary to infection of the primary tumor (10). In the present study, 19% of patients were positive for lymph node metastasis among 1016 included SCCP patients identified in the SEER database. Meanwhile, the Incidence of lymph node involvement can be upwards of 49% in intermediate-high risk tumors (pT1b, T2- T4) (23). Early metastatic spread to regional lymph nodes can be life-threatening. Guidelines had recommended lymphadenectomy because of concerns about the adverse effects of a delayed intervention on survival for penile cancer patients diagnosed with lymph node involvement. Therefore, the management of regional lymph nodes is very important for patient survival (24). Additionally, clinicopathological characteristics of tumors can be used to stratify patients and to prompt the inguinal lymph node dissection (ilND) performance (25). We constructed a predictive nomogram to evaluate the probability of lymph node metastasis in patients with M0 SCCP based on the SEER database.

Recently, A study established an NCDB-based nomogram to predict lymph node metastasis in penile cancer, showed that tumor grade, tumor lymphovascular invasion, and clinical lymph node status were all related to the increased incidence of lymph node metastases (26). Our research showed that the following five factors were independently associated with lymph node metastasis, including age, tumor grade, tumor size, T stage, and primary tumor site. All the above factors were selected for building the nomogram. Previous studies reported that model with AUC between 0.7 and 0.9 have moderate accuracy, indicating an acceptable degree of discrimination. In our study, this model had a calibration slope of 0.9 and a c-index of 0.776, indicating the good discrimination and effectiveness of the nomogram in predicting lymph node status. According to Youden's method, optimal cutoff values of the nomogram were 0.189, and the sensitivity, specificity associated with the 0.189 cut-offs were 79.5 and 34.2%, respectively. As we couldn't have both high sensitivity and high specificity, higher sensitivity is what we more needed considering that the purpose of our nomogram is to prevent false negativity. Historically, prophylactic inguinal lymph node dissection (ILND) has demonstrated a survival advantage in this population of patients, and failed detection of micrometastatic disease can have a significant impact on survival. However, contemporary philosophy dictates that subjecting all patients with the intermediate-risk disease to radical ILND carries an unacceptable risk of complications and long-term morbidity. By using the optimal cutoff value and the nomogram, we could predict the risk of lymph node invasion correctly in patients with different clinicopathological characteristics and determine the optimal management of lymph nodes in penile cancer. Regarding the five parameters included in this nomogram, the T stage had the highest discriminating power. Previous research showed that the incidence of lymph node involvement is 0–30% in patients with low-grade tumors (≤ T1a), while in patients with ≥T1b tumors or lymphovascular invasion, the incidence of lymph node metastasis is close to 50% (27). Among patients with advanced tumors, 50–70% of T2 tumor patients, and 50–100% of >T3 tumor patients have lymph node metastasis (28). Our study was consistent with previous research which reported that men with higher T stage were at higher risk of lymph node metastasis (29, 30). Consistent with previous studies (14, 31), we found that younger age was a high-risk factor of lymph node involvement, which may be related to human papillomavirus (HPV) infection. In pathogenic pathways involved in the development of penile carcinomas, about one-third of cases are associated with HPV infection (32), meanwhile, HPV infection has been found to have different age distribution characteristics (33). Our study corroborated those previous studies (12–14), with the addition of tumor size and primary tumor site as significant predictors. Tumor size ≥3 cm was significantly associated with an increased risk of lymph node involvement. Also, we analyzed the correlation between the primary site and clinicopathological characteristics of patients with penile squamous cell carcinoma and whether it is a risk factor for lymph node metastasis. Our research showed that tumors that occur in the body of the penis and overlapping lesions had a higher probability of lymph node metastasis.

In addition, recent research found that some biomarkers such as plasma C-reactive protein (CRP) and IGFBP2 levels were associated with higher tumor stages and lymph node metastasis (34, 35). Related predictive models can be further improved by including additional biomarkers.

Several limitations exist in our study. First, lymphovascular invasion (LVI) of the SEER database was recorded from 2010. Considering that the data in the database is incomplete, so we did not include it. As LVI was associated with increased rates of lymph node metastasis (13), including this predictive factor may improve the sensitivity and specificity of our nomogram. Besides, the SEER database was retrospectively collected and contains limited clinicopathologic data, central pathology review was not recorded, and there was no specific type of lymph node metastasis, so we cannot distinguish between the inguinal lymph node and pelvic lymph node metastasis. Generally, metastatic progression follows a predictable and stepwise pattern of invasion from the primary tumor to inguinal lymph basin before spreading to pelvic nodes and systemic dissemination, but distinguish pelvic from inguinal nodes is important to the identification of suitable surgical methods for nodal dissection. In addition, the predictive accuracy of nomograms should be tested through externally validated.

## Conclusion

In short, through retrospective analysis of 1,016 SCCP patients, this study established a new nomogram based on five independent risk factors to predict lymph node metastasis. The nomogram demonstrated well discrimination and effectiveness in predicting lymph node status. Although the prediction model has some limitations, the nomogram revealed the relationship between the clinicopathological characteristics of SCCP patients and the risk of lymph node metastasis. This tool will assist patients in counseling and guide treatment decisions for SCCP patients.

## Data Availability Statement

The original contributions generated in the study are included in the article/supplementary material, further inquiries can be directed to the corresponding author.

## Author Contributions

XZ designed the study. WZ, PG, and JG performed the literature research. Data were extracted by WZ, GL, and XW. WZ and XZ conducted the manuscript. All authors contributed to the article and approved the submitted version.

## Conflict of Interest

The authors declare that the research was conducted in the absence of any commercial or financial relationships that could be construed as a potential conflict of interest.
